# One size does not fit all: evaluating an intervention to reduce antibiotic prescribing for acute bronchitis

**DOI:** 10.1186/1472-6963-13-462

**Published:** 2013-11-04

**Authors:** Sara L Ackerman, Ralph Gonzales, Melissa S Stahl, Joshua P Metlay

**Affiliations:** 1 Department of Social and Behavioral Sciences, University of California San Francisco, San Francisco, CA, USA; 2 Division of General Internal Medicine, University of California San Francisco, San Francisco, CA, USA; 3 Geisinger Health System, Center for Health Research, Danville, PA, USA; 4 General Medicine Division, Massachusetts General Hospital, Boston, MA, USA

**Keywords:** Antibiotics, Clinician practice patterns, Patient education, Quality improvement

## Abstract

**Background:**

Overuse of antibiotics for upper respiratory tract infections (URIs) and acute bronchitis is a persistent and vexing problem. In the U.S., more than half of all patients with upper respiratory tract infections and acute bronchitis are treated with antibiotics annually, despite the fact that most cases are viral in etiology and are not responsive to antibiotics. Interventions aiming to reduce unnecessary antibiotic prescribing have had mixed results, and successes have been modest. The objective of this evaluation is to use mixed methods to understand why a multi-level intervention to reduce antibiotic prescribing for acute bronchitis among primary care providers resulted in measurable improvement in only one third of participating clinicians.

**Methods:**

Clinician perspectives on print-based and electronic intervention strategies, and antibiotic prescribing more generally, were elicited through structured telephone surveys at high and low performing sites after the first year of intervention at the Geisinger Health System in Pennsylvania (n = 29).

**Results:**

Compared with a survey on antibiotic use conducted 10 years earlier, clinicians demonstrated greater awareness of antibiotic resistance and how it is impacted by individual prescribing decisions—including their own. However, persistent perceived barriers to reducing prescribing included patient expectations, time pressure, and diagnostic uncertainty, and these factors were reported as differentially undermining specific intervention components’ effectiveness. An exam room poster depicting a diagnostic algorithm was the most popular strategy.

**Conclusions:**

Future efforts to reduce antibiotic prescribing should address multi-level barriers identified by clinicians and tailor strategies to differences at individual clinician and group practice levels, focusing in particular on changing how patients and providers make decisions together about antibiotic use.

## Background

Overuse of antibiotics for upper respiratory tract infections (URIs) and acute bronchitis is a persistent and vexing problem. Although the large majority of URIs and acute bronchitis cases are viral in etiology and therefore not responsive to antibiotics [1-3], more than half of all patients with these illnesses are treated with antibiotics every year in the U.S. [4,5]. Unnecessary antibiotic use for acute bronchitis is associated with increased risk of adverse drug events, higher overall health care costs, and increased patient and population level risk of drug resistant infections [6-8]. As a result, the rate of antibiotic use for patients with acute bronchitis is included as a HEDIS (Healthcare Effectiveness Data and Information Set) quality performance measure by the independent National Committee for Quality Assurance (NCQA) and reported by the vast majority of health insurers in the U.S. [5].

Attempts to reduce unnecessary antibiotic use have had mixed results. A combination of patient and physician education has been shown to help reduce antibiotic overuse for a variety of acute respiratory tract infections, including acute bronchitis [4,5], but levels of improvement have been limited, on average, to less than 20% absolute reduction across the study populations of physicians [9-12]. Some of the most effective interventions to date have been conducted in Europe. All have focused on improving clinicians’ communication skills, and the most successful have combined communication skills training with point-of-care testing for C reactive protein and the use of interactive booklets during consultations with patients [13-16]. Overall, antibiotic prescribing for acute bronchitis has been more resistant to change than for other acute respiratory infections, and the large majority (up to 90%) of patients diagnosed with acute bronchitis continue to be prescribed antibiotics [17-19].

Explanations for the intractability of antibiotic overuse for acute bronchitis include diagnostic uncertainty regarding the presence of alternative antibiotic-responsive illnesses (e.g., pneumonia), patient expectations in a societal context of consumer-oriented medicine and waning professional sovereignty among physicians, and time demands that favor expedient management decisions [20,21]. However, few studies have specifically explored whether these factors are barriers to effective implementation of interventions designed to reduce inappropriate antibiotic prescribing.

A three-armed cluster randomized trial of a multidimensional intervention strategy to reduce antibiotic prescribing for acute bronchitis was conducted at 33 primary care practice sites within the Geisinger Health System in Pennsylvania. Intervention sites received either print-based or electronic medical record (EMR)-based clinical decision support strategies in the form of treatment algorithms, in addition to patient education brochures and provider education seminars on appropriate prescribing practices.

Overall, the intervention resulted in a 12% absolute reduction in antibiotic prescribing (changes at EMR-based and print-based sites did not differ significantly), while prescribing rates increased slightly at the control sites [22]. Closer inspection of individual prescribing patterns showed that about 30% (N = 21) of clinicians decreased their prescribing rates substantially whereas the remaining two-thirds demonstrated variation in levels of prescribing that was no different from the control group. Based on available clinician characteristics, we did not find significant differences between the clinicians who changed their prescribing practices and those who did not.

To explore this clinician-level dichotomy in intervention response further, we investigated individual attitudes towards the intervention among clinicians at high and low performing sites, and considered these responses in the context of recent historical changes in clinician perceptions of antibiotic use more broadly. We conducted a mixed methods study using a computer-assisted telephone interviewing (CATI) instrument to assess physician attitudes towards antibiotic prescribing, and we compared the results with those of a similar survey conducted in 2000, in order to assess secular changes in physician attitudes towards antibiotic prescribing. We also asked open-ended questions to identify possible patient, clinician and practice-level reasons for the variability in the intervention’s impact, specifically focusing on reasons for the intervention’s failure to influence the prescribing behaviors of the majority of clinicians who were exposed to it.

## Methods

The cluster randomized trial involved 33 practices allocated equally to an EMR-based intervention, print-based intervention and control group [22]. For this survey study of a subset of practices, we sought to ensure equal representation of providers from practices with high and low antibiotic prescribing rates following the intervention. We invited all primary care clinicians (physicians, physician assistants, and nurse practitioners) practicing at the three practices with the highest antibiotic prescribing rates and the three practices with the lowest prescribing rates (based on the acute bronchitis HEDIS measure), for each of the print and EMR-based intervention arms of the trial, to participate in the study. High performing EMR-based intervention practices had an overall antibiotic prescribing rate of 33%, whereas low performing EMR-based intervention practices had an overall prescribing rate of 78%. At high performing print-based intervention practices, the prescribing rate was 55%, compared with 86% at low performing print-based intervention practices.

**Figure 1:**
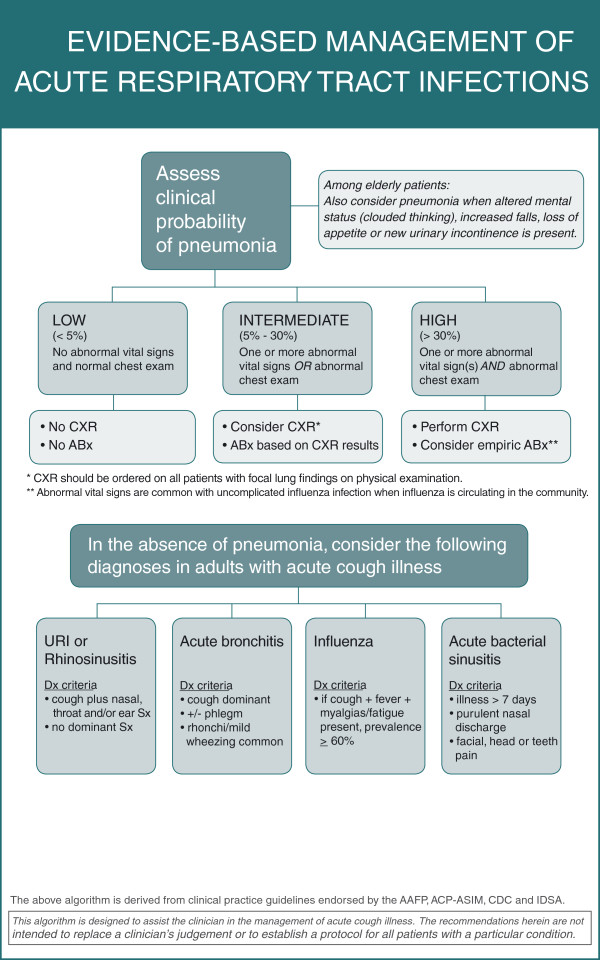
Exam room poster.

A total of 55 clinicians were recruited at the 12 intervention practices. At print-based practices, the intervention consisted of a poster depicting a diagnostic algorithm for cough illness that was placed in all examination rooms (see Figure 1). At EMR-based practices, a similar algorithm was programmed into the health system’s electronic medical record and clinicians were provided with an “order set” that recommended selected tests and treatments based on the specific diagnosis resulting from application of the algorithm (ie, acute bronchitis, sinusitis, URI, pneumonia).

All practices received patient education brochures with information about appropriate antibiotic use for cough and cold illness from the U.S. Centers for Disease Control [6]. The brochures were distributed by the nurse at the check-in desk of each clinic for any patient registering with a chief complaint of cough. No specific training was provided on the use of the brochures. Clinician “champions” were also assigned to each practice and invited to attend a training session led by R.G. and J.P.M., which included a lecture and small group discussion on the drivers of antibiotic prescribing in primary care settings and strategies for reducing patient expectation and antibiotic prescribing. The sessions were divided by EMR- and print-based intervention sites in order to include an orientation to the actual decision support tools.

After completing the training session, practice champions were asked to provide clinician education sessions and periodic feedback about prescribing practices for front line providers at their site. The content of these sessions included an educational slide set, provided by the study team and covering decision rules for appropriate antibiotic use, as well as tool-specific training. The sessions were held at the beginning of the intervention period and varied in size from one-on-one to small groups. Providers were aware that their prescribing practices were being monitored and that they would be invited to participate in a survey at the end of the study period. Providers were contacted by staff messaging within the EMR and by email and scheduled for the telephone survey at the end of the first year of the intervention.

Trained interviewers conducted the survey by telephone. Closed- and open-ended questions on three topics were included: 1) individual and societal consequences of antibiotic overuse; 2) barriers to improving antibiotic prescribing; and 3) perceived effectiveness of specific intervention components. Several of the questions in categories 1) and 2) were duplicated from a survey on attitudes towards antibiotic prescribing conducted with a national sample of primary care physician a decade earlier [23]. In addition, we added questions regarding perceived barriers to reducing antibiotic prescribing, including diagnostic uncertainty and patient expectations (see Additional file 1 for survey instrument). Closed-ended survey questions were measured on a five-point Likert scale and responses to open-ended questions were transcribed or paraphrased in writing by the interviewer. Responses were de-identified using a coding system with access restricted to the interviewers and authors.

The study was approved by the Geisinger Health System Institutional Review Board (IRB). For the clinical trial (reported separately), informed consent was not obtained from any patients or parents because the IRB determined that the quality improvement intervention focused on improving adherence to standards of care and did not require individual subject consent. The participant interviews with clinicians required verbal informed consent at the start of the interview. The IRB granted a waiver for written informed consent.

Quantitative survey data were summarized with frequency distributions, collapsing “strongly agree” and “agree” categories for each item. Unadjusted comparisons across groups were conducted with Chi-square test (or Fisher’s Exact test when cell sizes < 5 observations), comparing providers in both intervention arms and at high and low performing sites with each other and intervention providers with the historical control group. The constant comparative method was used for qualitative data analysis and integration of qualitative and quantitative results [24]. This process involved repeated reading and discussion of the survey responses by the study team in order to extract key themes. The triangulation of open- and closed-ended survey responses was conducted by comparing responses within and across individual respondents in order to discern patterns or associations. Survey responses for which associations between open- and closed-ended questions were not found are reported separately.

## Results

A total of 29 clinicians completed the survey (53% response rate), including 26 MD/DOs, 2 nurse practitioners, and 1 physician assistant. Nine respondents were from EMR-based intervention practices and 20 were from print-based intervention practices; 16 were from high performing sites and 13 were from low performing sites. There were 14 female and 15 male respondents, and on average they had practiced medicine at the Geisinger Health System for 8 years.

### Individual and community consequences of antibiotic overuse

Overall, 97% of respondents from the intervention practices agreed that antibiotic resistance is a major public health problem, and 93% believed that over-prescribing of antibiotics is a major cause of antibiotic resistance (Table 1). In the 2000 survey of primary care physicians, a similar majority (82%) of respondents agreed that antibiotic resistance is a major public health problem.

**Table 1:** Clinicians’ attitudes towards antibiotic prescribing over time

**Survey question**	**2000 (n = 400)% Agree**	**2010 (n = 29)% Agree**	**Difference (*P *** **value)**
Antibiotic resistance is a major public health problem.	82	97	0.0415
Over-prescribing antibiotics is a major cause of antibiotic resistance.	86	93	0.402
I am confident that the development of new and effective drugs will keep pace with the growing rate of antibiotic resistance.	21	17	0.813
Each individual decision to prescribe antibiotics has an impact on antibiotic resistance.	75	100	0.0005
I prescribe antibiotics more often thanI should.	36	66	0.003

Similarly, when asked to reflect on the importance of individual prescribing decisions, both their own and among clinicians more generally, all respondents in the current study concurred that each individual decision to prescribe antibiotics has an impact on community antibiotic resistance, compared with 75% agreement with the same statement 10 years earlier. An even more pronounced change was evident in responses to the statement, “I prescribe antibiotics more often than I should,” with 36% agreement in 2000 and 66% in 2010. On the other hand, it should be noted that in 2000 and 2010, 30% and 31% of respondents, respectively, reported that they themselves did not overprescribe antibiotics.

### Barriers to improving antibiotic prescribing

Participants in the trial identified several perceived barriers to practice change at multiple, interacting levels (Table 2). One of the most enduring was the belief that patients want antibiotics and will not be satisfied if they are not offered them. Most (79%) of the clinicians in the current survey reported that their patients really want and expect antibiotics, and will be dissatisfied if they don’t get them (72%).

**Table 2:** Clinicians’ perceived barriers to improving performance on the HEDIS (Healthcare Effectiveness Data and Information Set) acute bronchitis measure in 2010 (N = 29)

**Survey question**	**% agree**	**% disagree**
My patients really want and expect antibiotics when they come in.	79	21
My patients will be dissatisfied if they don’t get antibiotics.	72	14
It’s easier to just give the patient antibiotics than to explain that they don’t work.	38	55
I can’t always trust the CXR results to rule out pneumonia.	48	45
The randomized trials that found no benefit of antibiotics for acute bronchitis do not adequately represent patients in my practice.	21	62
I don’t have enough time with each patient.	41	52
I worry about possible medical liability if I don’t prescribe antibiotics and there’s a bad outcome.	34	34
Prescribing antibiotics for acute bronchitis is standard of care.	31	62

Several clinical and economic factors also appeared to serve as barriers to reducing inappropriate antibiotic prescribing. These include insufficient time with each patient, a problem that a notable minority (41%) of respondents reported was characteristic of their practice; a concern expressed by 34% of respondents about medical liability in the event that antibiotics are not prescribed and there is a bad outcome; and the challenging task of explaining medical evidence to patients. Indeed, a sizable proportion (38%) of respondents agreed that it is easier to give patients antibiotics than to explain that they don’t work.

Clinicians also reported diagnostic uncertainty and lack of generalizability of randomized controlled trial-based evidence as barriers to improving antibiotic prescribing. Specifically, 48% reported that they could not always trust chest x-ray results to rule out pneumonia, and 21% believed that randomized trials that found no benefit of antibiotics for acute bronchitis do not adequately represent patients in their practice.

### Clinicians’ responses to the intervention

Open-ended survey responses did not reveal meaningful differences by site (high vs. low HEDIS performance) in providers’ attitudes and perceptions of the intervention, but they did demonstrate considerable variation among individual clinicians in terms of how the intervention was received. Different opinions about the intervention may be related to individual clinicians’ perception of the problem of antibiotic overuse more broadly, and may point to possible reasons for the wide variability in the intervention’s effectiveness. This topic will be discussed in more detail below.

Both positive and negative assessments of both the print-based and EMR-based interventions highlight the importance of responsiveness to practice change initiatives in shaping how intervention components were received and acted on. For example, respondents appeared to fall into two categories of receptivity to practice change initiatives: those who think they cannot, or do not need to, change and those who appreciate being reminded of the need to change. Respondents in the first group reported that they were familiar with the message about reducing antibiotic prescribing prior to participating in the intervention, that antibiotic prescribing was already low at their site, or that education is unlikely to change patients’ expectations. Respondents in the second group were more likely to interpret the intervention as a kind of continuing education opportunity, as in the following statement: “It reminded you when antibiotic use would be more appropriate.” Interestingly, receptivity to the intervention was not correlated with any particular responses to the closed-ended survey questions.

In the following sections, we summarize in more detail the perceived weaknesses and strengths of the interventions.

### Intervention weaknesses

By far the most common clinician-reported obstacle to reducing antibiotic prescribing—one that most respondents insisted was not adequately addressed by the intervention—was patients’ expectations with regard to antibiotics. For example, both the on-site education session and patient brochure were critiqued by many respondents for their apparent inability to persuade patients (or help clinicians to persuade patients, in the case of the clinician education component) that antibiotics may not be the appropriate treatment for their condition. Participants emphasized that patients did not appear to read the brochure, did not “take in” its information, or “did not feel the brochure pertained to them.” In addition, as one clinician put it, a patient’s “mind has already been set [that they need antibiotics] before they see the doctor.” “They just want to leave with medication,” said another, suggesting that the brochures did not actually change patients’ assumptions about the appropriate use of antibiotics.

In addition, a majority of clinicians said that the EMR-based decision aid did not help them to influence patients’ expectations regarding antibiotics. “If the patient has in their mind that they absolutely need the antibiotic, they will leave with one,” said one clinician when asked about the EMR-based decision aid. Similarly, a minority of respondents did not approve of the print-based aid because they thought that it was not an effective medium for changing patients’ expectations. “Posters don’t change people’s minds,” said one clinician. Nor was it tailored to individual patients’ circumstances, as mentioned in this comment: “People think that the posters do not pertain to them.” These findings corroborate the closed-ended survey responses reported earlier, in which the majority of clinicians agreed that their patients want and expect antibiotics and will be dissatisfied if they do not receive them.

Another important factor in limiting participants’ response to the intervention was the perceived role that they, as individuals or groups of clinicians, play in antibiotic overprescribing and how amenable their routine clinical practices are to change. For example, some respondents suggested that overprescribing was not a problem at their site, and that the on-site education session presented by the clinic champion was therefore not necessary and might be more appropriate for clinics with “doctors giving out antibiotics like chewing gum.” Several participants reported that education on appropriate prescribing is unlikely to change “longstanding prescribing habits”, while others claimed that the clinicians attending the session were already familiar with the information presented. Although we did not find a significant correlation between these respondents and the nearly one third who disagreed with the statement “I prescribe antibiotics more than I should,” it is notable that both open and closed-ended survey questions revealed a subset of clinicians who do not feel that they are part of the problem of overprescribing.

Bearing out the results of the Likert-scale survey described above, the belief that evidence-based guidelines are not applicable to all patients, and uncertainty about correct diagnosis, are additional lenses through which respondents evaluated specific intervention components. For example, one clinician reported that the EMR decision tool and diagnostic algorithm could not be relied on because “guidelines don’t take into account the complexity of evaluating an individual patient.” Another clinician questioned the on-site education session content, stating that key indicators of pneumonia are not always apparent on an x-ray and that there may be reasons to be more aggressive with treatment that do not appear in the decision tool’s diagnostic criteria.

A final weakness of the intervention related to workflow. The majority opinion was that the requirements of the EMR-based decision aid, in particular, made it cumbersome and difficult to use, and that it undermined existing work processes. As one clinician put it, “It was too much for a simple order. I can just talk to a patient for five minutes and decide what is wrong, rather than go through the entire process and reach a different conclusion.” Several clinicians reported selectively using the electronic tool or not using it at all because it was “too long and comprehensive” or “too difficult to use.” In short, the clinical advantages of the EMR-based intervention did not outweigh its workflow disadvantages for the majority of respondents, particularly in comparison with the exam room poster. As one clinician reported about the EMR-based intervention “By the time you got to diagnose the problem it didn’t change anything for me.”

### Intervention strengths

The print-based decision aid was positively reviewed by the majority of surveyed clinicians for its usefulness when discussing diagnosis and treatment with patients. For example, the poster was praised for its simple visual depiction of the symptoms for which antibiotics are and are not indicated. Several clinicians described gesturing to the poster as a kind of “backup” expert when explaining prescribing decisions to patients. The poster was also reported to be a useful and accessible educational tool whose message about appropriate antibiotic use was made more persuasive by its clearly labeled affiliation with the medical center and the state department of health. Providing support for clinicians’ efforts to persuade patients that they do not need antibiotics may be particularly valuable in the exam room, particularly considering that the majority of our respondents believe that their patients will be dissatisfied if they leave without antibiotics.

Although the EMR-based decision aid was subject to more negative than positive comments, some clinicians reported that this component of the intervention made them follow “a more factual process” and “apply criteria” before prescribing antibiotics, and that it encouraged “good medical decision making”. Overall, the evidence-based, standardized approach to diagnosis and treatment that characterized the decision aid was perceived as improving clinical reasoning among some clinicians and undermining the clinical autonomy of others.

Finally, for some respondents the intervention did improve workflow. For example, several clinicians reported that the clinician education session and the print and EMR versions of the decision tool served as useful prompts or reminders about appropriate prescribing criteria, correct diagnostic codes for bronchitis, and the “true symptoms” of viral and bacterial infections.

## Discussion

Previous research has demonstrated that antibiotic prescribing is shaped by clinical, economic and social factors that coalesce in a “culture of prescribing”, whose norms enable the continuation of undesirable practices despite concerted efforts to change them [25-27]. Our results suggest that there has been some improvement in the culture of antibiotic prescribing for cough illness in the U.S. over the past 10 years, particularly in terms of a growing awareness among primary care clinicians of the scope and magnitude of antibiotic resistance, and of the contribution of individual prescribing decisions to the problem.

Nevertheless, the interventions evaluated here were not robust enough to make a more than modest impact on overall prescribing for acute bronchitis, and it was clear that they had a much stronger impact on some clinicians and clinical practice sites than others. Other studies have found significant variability in prescribing practices both geographically and among individual clinicians, which appears to be the result of clinical and non-clinical contextual factors that are resistant to change or beyond the scope of targeted interventions [28,29]. In our study, nearly a third of respondents did not believe that they overprescribed antibiotics even though their actual prescribing rates were on average as high as those of clinicians who report overprescribing (data not shown). A better understanding of the causes of clinicians’ (mis)perception is a requisite step in the design of a tailored intervention for this particular group, since the most effective approach for clinicians who acknowledge overprescribing and want to change their prescribing practices is likely to be different from approaches that will be successful with clinicians who do not acknowledge their own participation in the problem.

The belief that patients want and expect antibiotics was another key factor that appeared to undermine the intervention components’ perceived effectiveness, and that showed persistence over time in the 2000 and 2010 surveys. If patient expectations were thought to remain unaltered by the use of a decision aid (even if the aid specifically targeted the physician rather than the patient), then that intervention component was deemed less useful. Educational brochures, for example, were considered incapable of changing patients’ beliefs about antibiotic effectiveness for cough illness. Previous research suggests that printed educational materials for patients and/or providers have little effect on their own, but that they are more effective when used to guide communication between clinician and patient or when paired with verbal advice by a clinician [12,13,15,30-33]. This may be because social context, including the patient-clinician relationship, plays a more powerful role in shaping prescribing decisions than does information or algorithms that are abstracted from the interactive context of a medical decision [34,35]. Therefore, training clinicians to engage patients in a discussion about appropriate antibiotic use may be a more effective strategy for changing patient expectations than brochures or clinical practice guidelines alone.

Another example of the importance of patient-clinician interactions is the perception among respondents that the exam room poster was a more useful tool in discussions with patients than the EMR-based decision aid. From one point of view, the poster’s popularity as a persuasive communication device is at odds with its inability to influence overall prescribing rates more than the other intervention components. However, its limited success reinforces the need for strategies that assist clinicians with diagnosis and treatment discussions with patients. Indeed, prior research highlights the ambivalence experienced by clinicians as they attempt to balance their own “individual best practice” criteria with patient expectations, in particular when patient desires conflict with professional opinion [36,37]. Future attempts to change prescribing practices should engage both patients and clinicians during the clinical encounter, and should be flexible enough to assist and enable clinicians with different communication styles and degrees of experience to anticipate and respond to a range of explicit or tacit requests for antibiotics from patients [38].

Our findings also suggest variability in clinicians’ beliefs about how to navigate the complex intersection of evidence-based criteria and the specificity of an individual patient’s condition. For some of our participants this meant that their own clinical judgment should be able to overrule evidence-based practice guidelines, policies, or decision aid recommendations. For others these same guidelines and decision aids were perceived as an important source of assistance and professional improvement, but only if they did not interfere with clinical or administrative procedures. For nearly all respondents, a tool that was perceived as unwieldy, time-consuming, or undermining of existing work processes, was unlikely to be widely adopted. In the future, decision support tools for antibiotic prescribing should strike a balance between promoting practice change and supporting individual clinicians’ modes of balancing clinical and population-level evidence.

Our study did not have a large-enough sample size to detect whether differences in perceived barriers to improving antibiotic prescribing help explain differences in clinicians’ responses to the intervention. Nor were we able to discern whether variations in perceived barriers accounted for prescribing differences at high and low performing intervention sites. Moreover, the survey responses in the table were drawn from two distinctly different studies. The small sample size, geographic specificity, and single institutional affiliation of our study’s participants may not be generalizable to primary care clinicians nationally, whereas the 2000 survey was conducted with a larger national sample of internists and family practice physicians. In addition, the 2000 survey was conducted anonymously by mail, whereas our survey was conducted on the phone by a research assistant halfway through an intensive, hands-on intervention, so the responses reported here may have been influenced by exposure to intervention materials and staff.

## Conclusions

A growing literature on strategies to reduce the overuse of antibiotics demonstrates that multifaceted interventions tend to be the most successful [12,39]. Our results also suggest that efforts to reduce antibiotic prescribing may be more effective if they intervene directly in the clinical encounter and attend to key differences among individual clinicians and group practices. A nuanced assessment of the reasons for practice variation – both among individual clinicians and between group practices – could be used to determine which change strategies would be most appropriate for groups of clinicians with similar prescribing patterns, and how these interventions should be adjusted to fit the specific clinical and social contexts in which patients and clinicians come together and make decisions [40].

To work towards this goal, we recommend a more tailored approach to the problem of overprescribing—one that draws on social and behavioral science theory and methods to guide research and intervention design. This would include: a) in-depth, qualitative and quantitative assessments of how context—specifically norms, values, relationships and resources, produce variations in the impact of interventions aiming to improve antibiotic prescribing; b) adapting intervention design to different groups of providers using, for example, the social marketing strategy of “audience segmentation” [41]; and c) building flexibility and responsiveness into the design of change strategies so that interventions can be adjusted mid-stream based on clinician and patient feedback.

## Competing interests

SA, JM and MS declare that they have no competing interests, financial or otherwise. RG serves as Medical Advisor to Phreesia, Inc. He declares that he has no other competing interests, financial or otherwise.

## Authors’ contributions

SA participated in data analysis and drafted the manuscript. RG designed the study, participated in data analysis, and helped to draft the manuscript. MS carried out data collection and helped to draft the manuscript. JM designed the study, participated in data analysis, and helped to draft the manuscript. All authors read and approved the final manuscript.

## Authors’ information

SA is a medical anthropologist whose research focuses on the social and cultural dimensions of new medical technologies, and how these devices and systems influence patient-clinician interactions and medical decision making.

RG is a general internist whose research focuses on improving health care delivery and quality through multi-faceted, patient-centered intervention strategies. He serves as Associate Chair of Ambulatory Care and Clinical Innovation, and directs the Implementation Science Program at UCSF.

MS is a research project manager whose experience focuses on developing and managing health information technologies to improve quality and outcomes of care. Specifically, technologies that support patient decision-making, provider decision support, and patient-provider communication.

JM is a general internist whose research focuses on studying the relationship between treatment and outcomes for patients with acute respiratory tract infections and translating that research into practice. He directs the Center for Healthcare Improvement and Patient Safety at the University of Pennsylvania, which is dedicated to supporting research and research training in quality improvement and implementation science.

## Pre-publication history

The pre-publication history for this paper can be accessed here:

http://www.biomedcentral.com/1472-6963/13/462/prepub

## Supplementary Material

Additional file 1 CDC Acute Bronchitis Interview Script. Click here for file
