# Supplementary material for: One size does not fit all: evaluating an intervention to reduce antibiotic prescribing for acute bronchitis
Source: BMC Health Serv Res. 2013 Nov 4;13:462. doi: 10.1186/1472-6963-13-462 (PMC4228248; doi:10.1186/1472-6963-13-462)
Supplement: Additional file 1 — CDC Acute Bronchitis Interview Script. [file 1472-6963-13-462-S1.doc]

**CDC Acute Bronchitis Interview Script**

**Name of Practice:**

**Name of Participant:**

**Job Title:**

**Years with Geisinger:**

The CDC-sponsored Acute Bronchitis Project is a collaboration between Geisinger Health System, the University of Pennsylvania and the University of California, San Francisco. Briefly, the acute bronchitis project is a quality improvement project that is aimed at reducing the overuse of antibiotics for adults with uncomplicated acute bronchitis—a new HEDIS measure. Today you will be asked questions about your knowledge of quality improvement efforts at your practice, and your awareness and experiences with the acute bronchitis project.

Before we start, I need to go over some administrative issues. Your participation in this focus group is completely voluntary and confidential. Your responses will be combined with other interviewees, and no identifiable information about you will be stored or reported.

Do you/anyone have any questions or concerns?

**1**) Over the past 12 months, do you recall any activities at your practice aimed at reducing the overuse of antibiotics for bronchitis or cough illness?

1.A. Yes or No. If Yes, continue below. If No, go to next question.

1.A.1. Do you remember a lecture/ talk on this topic?

1.A.2. Do you remember exam room posters on appropriate antibiotic use?

1.A.3. Do you remember waiting room brochures on this topic?

1.A.4. Do you remember a Best Practice Alert for cough illness?

1.A.5. Do you remember a Smartset order for acute cough illness?

1.B. Of the interventions you remember, which ones do you believe were effective?

1.B.1. lecture/ talk on this topic?

1.B.1.a. why?

1.B.2. exam room posters on appropriate antibiotic use?

1.B.2.a. why?

1.B.3. waiting room brochures on this topic?

1.B.3.a. why?

1.B.4. Best Practice Alert for cough illness?

1.B.4.a. why?

1.B.5. Smartset orders for acute cough illness?

1.B.5.a. why?

1.C.1. Who in your practice was responsible for leading this project?

*(If answer is unknown, document this response and then provide the respondent with the name of the physician champion at their site).*

1.C.1.a. On a scale of 1 to 5, where 1 is “not at all effective”, 3 is “somewhat effective” and 5 is “extremely effective”, how effective do you think this person was?

1.C.1.b.1 Is there anything that he/she could have done better?

**2) I would like to ask you to help us identify the barriers to improving antibiotic use in your practice. Please let us know how much you agree or disagree with the following statements as potential barriers to improving antibiotic use in your practice, where 1=strongly disagree, 5=strongly agree and 3=neutral.**

2.A. My patients really want and expect antibiotics when they come in.

2.B. My patients will be dissatisfied if they don’t get antibiotics.

2.C. I can’t always trust the CXR results to rule-out pneumonia

2.D. The randomized trials that found no benefit of antibiotics for acute bronchitis do not adequately represent patients in my practice.

2.E. I don’t have enough time with each patient.

2.F. It’s easier to just give the patient antibiotics than to explain that they don’t work.

2.G. Prescribing antibiotics for acute bronchitis is standard of care.

2.H. I worry about possibly medical liability if I don’t prescribe antibiotics and there’s a bad outcome.

**3) Next I would like to ask you to give us your opinion about some issues regarding the use of antibiotics for the management of respiratory tract infections. Please let us know how much you agree or disagree with the following statements, with 1=strongly disagree, 5=strongly agree and 3=neutral.**

3.A. I am confident that the development of new and effective drugs will keep pace with the growing rate of antibiotic resistance.

3.B. Antibiotic resistance is a major public health problem.

3.C. I prescribe antibiotics more often than I should.

3.D. Each individual decision to prescribe antibiotics has an impact on antibiotic resistance.

3.E. Over-prescribing antibiotics is a major cause of antibiotic resistance.

3.F. Physicians should consider the needs of the individual patient when prescribing an antibiotic.
